# COVID-19 infection in patients with multiple myeloma: a German-Chinese experience from Würzburg and Wuhan

**DOI:** 10.1007/s00277-020-04184-2

**Published:** 2020-07-18

**Authors:** Xiang Zhou, Tao Bai, Katharina Meckel, Jun Song, Yu Jin, K. Martin Kortüm, Hermann Einsele, Xiaohua Hou, Leo Rasche

**Affiliations:** 1 Department of Internal Medicine II, University Hospital Würzburg, Julius-Maximilian University of Würzburg, Oberdürrbacher Street 6, D-97080 Würzburg, Germany; 2 Department of Gastroenterology, Union Hospital Wuhan, Tongji Medical College, Huazhong University of Science and Technology, 1277 Jiefang Avanue, Wuhan, 430030 China

Dear Editor,

The Chinese colleagues from Wuhan reported for the first time the novel coronavirus (COVID-19), which caused severe acute respiratory syndrome [1]. Currently, COVID-19 continues to spread worldwide and is affecting all fields of health care including patients with malignant diseases. The plasma cell neoplasm multiple myeloma (MM) represents the second most common hematological malignancy in adults, which is characterized by secondary immune dysfunction and usually causes infectious complications, especially of the respiratory tract [2–4]. At present, there are only few reports on COVID-19 infection in patients with hematological malignancies including MM, from China, the USA, and the UK [5–8]. Generally, experience with COVID-19 in MM is still very limited, and there may be regional differences in disease severity. Therefore, we conducted this study of MM patients infected by COVID-19 and compared the clinical features of patients from Germany and China.

We retrieved and analyzed the data of MM patients with laboratory confirmed COVID-19 infection at two prominent hematology centers in Wuhan and Würzburg (Union Hospital of Tongji Medical College, Huazhong University of Science and Technology, Wuhan, China, and University Hospital of Würzburg, Würzburg, Germany) as of 9 June 2020. This study was performed in accordance with the Declaration of Helsinki as revised in 2013 and with national ethical standards at both centers.

We summarized patients’ characteristics, treatment, and outcome in Table 1. In total, we identified five Caucasian patients from Würzburg (Nos. 1–5) and three Asian patients from Wuhan (Nos. 6–8). The majority of the patients were male (*n* = 5, 63%), and the median age at COVID-19 diagnosis was 57 (range 39–83 years). Only one patient (No. 2) presented high-risk cytogenetics, i.e., t(4;14). Three patients (Nos. 5, 7, and 8) had newly diagnosed (ND) MM, and two of them (Nos. 5 and 8) were therapy naïve at diagnosis of COVID-19. One patient (No. 7) from Wuhan was receiving the second cycle of VTD (bortezomib, thalidomide, and dexamethasone) as frontline therapy. High-dose melphalan with autologous stem cell transplant (SCT) was performed in three patients (Nos. 1–3), all from Würzburg. At the time point of COVID-19 diagnosis, three patients (Nos. 1, 3, and 4) were treated with daratumumab-containing regimens. In Wuhan, a patient with extramedullary progression (No. 6) received leukapheresis to prepare for a salvage chimeric antigen receptor T-cell (CAR-T) therapy, and this patient was hospitalized in the hematology department until 31 January 2020. The three patients from Wuhan were infected by COVID-19 in January or February 2020, while the Würzburg patients were diagnosed in March or April 2020. Due to COVID-19 infection, anti-MM treatment was discontinued in all the patients. Notably, two patients (Nos. 3–4) in Würzburg showed no COVID-19 symptoms, and the other three patients (Nos. 1, 2, and 5) exhibited only mild symptoms such as fever, cough, and nausea, which did not require an intensive care unit (ICU) admission. Three patients (Nos. 2, 3, and 5) did not receive any COVID-19 treatment, and all five patients in Würzburg recovered. In contrast, two patients (Nos. 6–7) from Wuhan developed severe respiratory syndrome, so mechanical ventilation and circulatory support were needed. The patient No. 7 who was receiving the frontline therapy with VTD also had an elevated procalcitonin value (30.05 ng/ml), suggesting an additional bacterial infection, and, despite maximal medical care, this patient died due to acute respiratory failure. Interestingly, approximately 3 weeks after diagnosis, as the patient No. 6 was discharged and the swab was also negative for COVID-19, both COVID-19 IgM and IgG were tested negative in this patient. In four patients from Würzburg, we also performed COVID-19 antibody test after recovery, and three of them (Nos. 1, 2, and 5) showed positive IgG, while one patient (No. 3) did not develop IgG or IgM against COVID-19. This finding suggested inadequate humoral immune response in MM patients, probably due to secondary immune deficiency caused by the treatments or the disease itself. Unfortunately, the data of COVID-19 antibody test were not available in the other patients. Of note, the patient No. 6 was hospitalized until the end of January 2020, and 2 weeks later, he developed symptoms and was diagnosed with COVID-19 infection. This observation suggested that it might be a nosocomial infection in this patient. After recovery, two patients from Würzburg received MM therapy, i.e., lenalidomide maintenance in one patient and DARA-VRCD (daratumumab, bortezomib, lenalidomide, cyclophosphamide, and dexamethasone) in another patient with NDMM.

**Table 1:** Summary of patients’ characteristics, treatment and outcome

Patient	Site	Age at COVID-19	Gender	MM subtype	High-risk cytogenetics*	Prior lines of therapy	Pretreatment	Current therapy	EMD	Time since MM diagnosis, months	Date of COVID-19 diagnosis	COVID-19 symptoms	Pulmonary infiltration	CRP, mg/l	PCT, ng/ml	COVID treatment	ICU admission	Mechanical ventilation	Circulatory support	Survival status at discharge	Retreatment after COVID-19	COVID-19 antibody after recovery
1	Würzburg	53	Male	IgG	No	2	PI, IMiD, ASCT, DARA	DRD	None	142	11.04.2020	Cough, fever	Yes	50	0.10	IVAB,HCQ	No	No	No	Alive	No	IgG positive
2	Würzburg	50	Male	IgG	Yes	1	PI, IMiD, ASCT	Lenalidomide maintenance	None	22	14.03.2020	Cough, fever, myalgia	Yes	40	0.10	None	No	No	No	Alive	Lenalidomide maintenance	IgG positive
3	Würzburg	70	Male	IgG	No	2	PI, IMiD, ASCT, DARA	DARA-VRD	None	58	16.04.2020	Asymptomatic	No	10	0.08	None	No	No	No	Alive	No	IgG, IgM negative
4	Würzburg	83	Female	IgG	No	4	PI, IMiD, DARA	DVD	None	94	23.04.2020	Asymptomatic	Yes	48	0.10	IVAB	No	No	No	Alive	No	NA
5	Würzburg	79	Female	IgA	No	0	None	n.a.	None	NA	01.04.2020	Nausea	No	29	0.05	None	No	No	No	Alive	DARA-VRCD	IgG positive
6	Wuhan	39	Male	LC	NA	4	PI, IMiD	S/P CAR-T apheresis	CNS, pleura, lung, para-medullary lesion	30	17.02.2020	Cough, fever, vomiting, seizure	Yes	13	0.21	IVAB	Yes	Yes	Yes	Alive	CAR-T in preparation	IgG, IgM negative
7	Wuhan	53	Female	IgA	NA	1	PI, IMiD	VTD	Para-medullary lesion	3	25.01.2020	Fever, fatigue	Yes	106	30.05	IVAB, arbidol, IFN-α, oseltamivir	Yes	Yes	Yes	Dead	NA	NA
8	Wuhan	61	Male	IgG	NA	0	None	n.a.	None	NA	23.01.2020	Chest pain	Yes	108	0.07	IVAB, arbidol, IFN-α, oseltamivir	No	No	No	Alive	NA	NA

CAR-T, chimeric antigen receptor T-cells; ASCT, autologous stem cell transplant; CNS - central nervous system; CRP, C-reactive protein; DARA, daratumumab; DARA-VRD, daratumumab, bortezomib, lenalidomide, dexamethasone; DARA-VRCD, daratumumab, bortezomib, lenalidomide, cyclophosphamide, dexamethasone; DRD, daratumumab, lenalidomide, dexamethasone; DVD, daratumumab, bortezomib, dexamethasone; EMD, extramedullary disease; HCQ, hydroxychloroquine; ICU, intensive care unit; IFN-α, interferon-α; Ig - immunoglobulin; IMiD, immunomodulatory drug; IVAB, intravenous antibiotics; LC, light chain; MM, multiple myeloma; NA, not available; PCT, procalcitonin; PI, proteasome inhibitor; VTD, bortezomib, thalidomide, dexamethasone; *at least one of the following: t(4;14), t(14;16), del(17p)

He et al. reported 13 COVID-19 patients with different hematological malignancies including three MM patients, and, in the entire group, the case fatality rate was 62% [5]. Additionally, a study from the USA demonstrated the severity and high mortality of COVID-19 infection in MM patients, especially in African American [8]. In another larger study from the UK, the mortality for COVID-19 was even as high as 54.6% (41/75) in MM patients, which was significantly higher than the general population [7]. Importantly, both studies suggested that patients with NDMM had a higher mortality rate, which might be related with MM-induced immune suppression and risk of infection caused by bacteria, fungi, and other viruses [7, 8]. A lower risk for developing hyper-inflammatory immune responses in late-stage patients might be an alternative explanation. Dufour et al. recently reported the experience from Belgium in this Journal (published on 23 June 2020), which also demonstrated a high mortality of 35% caused by COVID-19 infection in MM patients, and patients with immigration background especially North-Africans showed the worst survival outcome. However, the majority of the patients who passed away (5/7, 71%) had progressive MM at COVID-19 [9]. In our cohort, the only patient who died was a patient with NDMM receiving frontline therapy, and she showed signs of additional bacterial infection. Surprisingly, the MM patients in Würzburg did not present any signs of severe COVID-19 infection. Other than Wuhan where COVID-19 was reported for the first time, the pandemic had been announced in Europe, and in Germany, the lockdown came relatively early in comparison with the other countries. In addition, as two out of five patients in our cohort did not show positive IgM or IgG for COVID-19 after recovery, the role of serologic test to identify asymptomatic cases in MM should be further evaluated. Currently, the COVID-19 pandemic worldwide is still not under control, and there is still no causal treatment or vaccine for COVID-19, so we probably have to face this virus for longer time than expected. Strategies such as rational local organization, local disinfection, and hygiene maintenance, training of health workers and patients represent important steps to prevent nosocomial infection, which are also applicable for the time after the pandemic [10]. Importantly, MM patients with COVID-19 infection need close monitoring for severe COVID-19-related complications, and early intervention may be life-saving for the patients [11].
